# Gene expression study in the siRNA based aniridia cell model and in primary aniridia limbal epithelial cells following duloxetine and ritanserin treatment

**DOI:** 10.1371/journal.pone.0324829

**Published:** 2025-06-10

**Authors:** Shweta Suiwal, Tanja Stachon, Zhen Li, Marta Corton, Mahsa Nastaranpour, Ning Chai, Maryam Amini, Berthold Seitz, Fabian N. Fries, Thomas Tschernig, Nóra Szentmáry

**Affiliations:** 1 Dr. Rolf M. Schwiete Center for Limbal Stem Cell and Aniridia Research, Saarland University, Homburg/Saar, Germany; 2 Departamento de Genética, Hospital Universitario Fundación Jiménez Díaz Madrid España, Área de Genética & Genómica, Instituto de Investigación Sanitaria - Fundación Jiménez Díaz - Universidad Autónoma de Madrid (IIS-FJD, UAM) Madrid España; Centro de Investigación en Red de Enfermedades Raras (CIBERER), ISCIII Madrid España,; 3 Department of Ophthalmology, Saarland University Medical Center, Homburg/Saar, Germany; 4 Institute for Anatomy and Cell Biology, Saarland University, Homburg/Saar, Germany; Cedars-Sinai Medical Center, UNITED STATES OF AMERICA

## Abstract

Progressive aniridia associated keratopathy is worsening visual acuity of congenital aniridia subjects lifelong. Restoration of PAX6 expression in PAX6 haploinsufficient limbal epithelial cells could be one therapeutic option. In a previous study using aniridia-like CRISPR/Cas9 genome-edited corneal epithelial cells, the antipsychotic drugs duloxetine and ritanserin increased PAX6 mRNA and protein expression. Our purpose was to investigate the effect of duloxetine and ritanserin on cultured primary limbal epithelial cells (pLECs) without and with PAX6 knockdown. pLECs were isolated from 11 aniridia patients and corneoscleral rims of 8 healthy human donors and were treated with 5 µM duloxetine or ritanserin for 24 hours. In addition, pLECs were transfected with small interfering RNA (siRNA) (PAX6 knockdown) in the siRNA-based aniridia cell model and were also treated by 5 µM duloxetine or ritanserin for 24 hours. Gene and protein expression were analyzed using qPCR and Western blot. In both primary aniridia limbal epithelial cells and the siRNA-based aniridia cell model, the expression of *PAX6* at the transcriptional or translational level did not show significant changes through duloxetine or ritanserin treatment (p > 0.5). The target genes of *PAX6* such as *KRT3, KRT12, DSG1*, *ALDH1A1, ADH7*, *FABP5*, *ABCG2* also did not change significantly (p ≥ 0.2). Our study shows that primary cultures of limbal epithelial cells from both aniridia patients and healthy donors were unresponsive to drug treatment. Therefore, our data suggest that different aniridia cell models or cell culture conditions exhibit varying responses to duloxetine and ritanserin. The use of in vivo models could further enhance our understanding of duloxetine and ritanserin treatment in aniridia-associated keratopathy.

## Introduction

Congenital aniridia is a rare panocular disease that affects the cornea, iris, anterior chamber, lens, retina, and optic nerve head. It has been well accepted that in most cases congenital aniridia is caused by a dominantly inherited heterozygous mutation in paired box 6 (*PAX6*) gene [1–4]. The transcription factor PAX6 is essential for normal eye development and function, and it is expressed in healthy mature corneal and conjunctival epithelial cells as well as in regions of the olfactory epithelium, pancreas and central nervous system [5–9]. The majority of the *PAX6* gene mutations are nonsense mutations that lead to premature stop codon in exons and non-coding regions of the *PAX6* gene. These mutations affect most of the time one copy of the gene, leading to *PAX6* gene haploinsufficiency [1,3].

PAX6 haploinsufficiency causes abnormal eye development, limbal epithelial stem cell insufficiency and corneal opacification, which is known as aniridia associated keratopathy (AAK) [10–13]. In most aniridia cases, the AAK-related corneal neovascularization and pannus formation is the main reason of the progressive visual loss of the patients. AAK mostly begins early in infancy and may progressively lead to untransparent corneal opacity with severe visual impairment and potential blindness for adulthood [10–15]. Therefore, AAK is considered as a prime target of therapies in congenital aniridia subjects.

Due to the *PAX6* mutation, AAK is characterized by loss of the limbal epithelial stem cell niche in a time dependent manner. Therefore, identification and testing of drugs that can rescue phenotypes due to *PAX6* haploinsufficiency can be crucial. In AAK, the insufficient PAX6 protein amount is also responsible for the severity of the congenital aniridia phenotype, and the severity could be directly correlated with the PAX6 mutational status [3]. Thus, increasing the expression of *PAX6* can potentially alleviate the progression of AAK.

Although various potential treatment options have already been suggested to treat AAK in humans, such as for example MiniPromoters for ocular gene therapy [16], all had either too high risks for the patients or possessed low success rate. In an approach to manipulate PAX6 mutational status, it has been reported that adenine base editor (ABE8e), a CRISPR enzyme encapsulated as lipid nanoparticles of ribonucleoprotein (LNP-RNP), mediated the genetic sequence editing of *PAX6* variants for aniridia in humanized mouse embryonic stem cells. This CRISPR based strategy corrected the *PAX6* variant and thereby rescued PAX6 protein expression [17]. However, through this strategy, only the most common congenital aniridia variant was corrected, which is present in about 20% of the cases. Additionally, the direct translation of this approach to humans is disadvantageous as CRISPR enzymes could have off targets in humans [17].

Likewise, another molecule, ataluren can be used as therapeutic nonsense suppression agent that increases PAX6 protein levels in *Pax6*^*Sey/+*^ mice by delivering postnatally the ataluren in a dose and time dependent manner [18]. Nevertheless, the aniridia mouse models do not completely recapitulate the aniridia phenotypes that are observed in human patients, thereby decreasing the chance of these therapeutic agents to be considered as treatment options. In addition, the possible toxic effects of these therapeutic agents are also a concern to translate into clinical use. Nonetheless, all these approaches require demonstration of safety for clinical translation [18,19].

Cornea is a densely innervated tissue and contains nerves derived from the peripheral nervous system and produces serotonin in addition to the classical neurotransmitters [20,21]. Interestingly, serotonin levels were significantly higher in aqueous tear deficiency and dry eye symptomatic patients, than in controls [22]. However, the link between *PAX6* and the anti-psychotic drugs is still not clearly understood. Nevertheless, that *PAX6* is not only responsible for eye development but is also expressed in neural stem cells and progenitor cells, suggests a relationship between PAX6 and the anti-psychotic drugs (duloxetine and ritanserin) [23,24]. Roux et al. in 2018 demonstrated that the FDA approved antipsychotic drugs duloxetine and ritanserin increased PAX6 mRNA and protein expression in aniridia-like CRISPR/Cas9 genome edited corneal epithelial cells [25]. Duloxetine is a norepinephrine and serotonin reuptake inhibitor, which is an approved antidepressant drug, used for patients with generalized anxiety disorder and major depressive disorder [26]. Duloxetine increases dopamine levels within the prefrontal cortex by inhibiting norepinephrine transporters [27]. Duloxetine administration for relieving neuropathic pain or modulating brain derived neurotrophic factors suggested duloxetine’s potent effect on serotonin levels or on serotonin transporter, in several animal models [28,29]. Duloxetine use has been also suggested for chemotherapy-induced neuropathies (non-FDA approved) [30,31]. Ritanserin is a serotonin reuptake inhibitor (5-HT_2_ antagonist), a potential drug for schizophrenia treatment, as well as sleeping disorder [25,32]. Although the exact mechanism how ritanserin works is largely unknown, Olmez et.al. have demonstrated that ritanserin inhibits diacylglycerol kinase alpha (DGKα) and thereby affecting NF-κB in mesenchymal cells [33].

In this study, we aimed to evaluate the efficacy of duloxetine and ritanserin on primary limbal epithelial cells. However, due to the low number of aniridia patients, the availability of primary aniridia limbal epithelial cells to develop therapeutic options is limited. To address this, we first established a small interfering RNA (siRNA)-based primary aniridia cell model, utilizing *PAX6* gene knockdown to mimic *PAX6* deficiency in primary limbal epithelial cells, *in vitro* [34]. The strength of this approach lies in its potential to identify target genes influenced by *PAX6* that may contribute to AAK pathogenesis therefore, making it a suitable model to test potential AAK drugs capable of alleviating PAX6 protein expression. While all the cell models have their limitations, the use of primary cell cultures, though with limited availability, provides valuable insights that complement results from other experiments. In this study, we analyzed the PAX6 gene and its target gene expressions in control siRNA and *PAX6* siRNA (*PAX6* knockdown) transfected primary limbal epithelial cells, as well as in primary aniridia limbal epithelial cells following treatment with duloxetine and ritanserin.

## Materials and methods

### Ethical considerations

Our study followed the regulations of the Declaration of Helsinki and was approved by the Ethics Committee of Saarland/Germany (No 21/21). Limbal biopsies from aniridia patients, limbal biopsies and corneoscleral rims from healthy donors were obtained from 15^th^ February 2021–19^th^ March 2024 from the Klaus Faber Center for Corneal Diseases including Lions Eye Bank (Tables 1–2) for this study. Informed written consent was obtained from patients with aniridia. In case of one minor aniridia patient, informed written consent was obtained from the patient’s parents or guardians.

**Table 1:** Congenital aniridia donors, used in the study. Aniridia associated keratopathy (AAK) was graded according to Lagali et al [3].

Gender	Age (years)	Mutation type	Functional consequence (predicted)	Affected region	DNA change	Protein change	AAK grade
F	28	PTC	NMD Inducing	Exon 9	c.781C > T	p.(Arg261*)	5
F	16	Chromosomal	WAGR	n/a	46,XX del	p13-ter	4
F	16	Deletion	n/a	Exon 11–15 + ELP4 Exon 9	PAX6	n/a	4
F	57	Deletion	NMD inducing	n/a	c.33delC	p.Gly12Valfs*19	4
M	43	Splice site	Removing the stop codon	Intron 12	(c.1226-2A > G)	n/a	4
F	6	C-terminal extension	n/a	Exon 13	c.1267dupT	p.*423Leuext*36	4
F	57	n/a	n/a	Intron 10	c.916 + 1G > C	n/a	3
M	41	Splice site	n/a	Intron 5	c.142-3C > G	n/a	3
M	37	Nonsense	n/a	Exon 11	c.949C > T	p.(Arg317*)	3
F	25	Nonsense	n/a	Exon 4	c.4C > T	p.(Gln2*)	3
M	27	Nonsense-Mutation,PTC	truncated protein	Exon 10	c.829C > T	p.(Gln277*)	3

NMD: Nonsense-mediated RNA decay; n/a: not available.

**Table 2:** Healthy donors (limbal epithelial cells (LEC) and control (CTRL) biopsies) used in the study.

Sample number	Age (years)	Gender
LEC 1	75	Female
LEC 2	93	Female
LEC 3	77	Female
LEC 4	98	Female
LEC 5	63	Male
LEC 6	85	Female
LEC 7	n/a	Male
LEC 8	n/a	Female
LEC 9	n/a	Female
LEC 10	n/a	Male
LEC 11	n/a	Male
LEC 12	60	Male
LEC 13	73	Female
LEC 14	n/a	Male
LEC 15	n/a	Female
LEC 16	79	Male
LEC 17	n/a	Female
LEC 18	71	Male
LEC 19	85	Female
LEC 20	65	Male
LEC 21	65	Male
CTRL biopsy	90	Male

n/a: not available (To determine the gender of some donors, genotyping of LEC 7–22 has been performed by analysing *SRY* and *NLGN4X/Y* gene expression (**Table 3**) [35]).

### Cell culture

#### Primary healthy and aniridia limbal epithelial cells.

For cell culture, primary limbal epithelial cells (pLECs) were isolated from corneoscleral rims of healthy donors or limbal biopsies from aniridia patients as described previously [36,37].

First, 1.5 mm diameter limbal pieces were punched out from the limbal region of corneoscleral rims. These limbal pieces and biopsies were incubated in 100 µl collagenase A (4 mg/ml) (Roche Pharma AG, Basel, Switzerland) in 700µl Keratinocyte serum free medium (KSFM, Cat. Nr. 17005042, Thermo Fisher, Gibco, Life Technologies, Paisley, UK), supplemented by 50 µg/ml Bovine Pituitary Extract (BPE), 5 µg/ml Epidermal Growth Factor (EGF) (Gibco, Life Technologies, Paisley, UK) and 100 U/ml Penicillin/Streptomycin (P/S) (Sigma Aldrich, Germany) overnight at 37 °C. Then, the limbal pieces were pipetted up and down and were loaded onto a 20 µm Cell Tricks filter to retain the epithelial cell clusters and remove the fibroblasts. After washing the filter with 10 ml phosphate buffer saline (PBS), the retained cell clusters, attached to the filter were dissolved using 1.5 ml trypsin-EDTA-solution (Sigma-Aldrich GmbH, Deisenheim, Germany). Dulbecco’s Modified Eagle Medium (DMEM) with 5% FCS and 100 U/ml P/S was used to stop the trypsin reaction. Cell suspension was centrifuged, cell pellet was resuspended in 3 ml KSFM and the cells were seeded into a single well of a 24-well plate. Medium was exchanged every 3 days and after reaching 90% confluence, LECs were passaged into one well of a 6-well plate using 500 µl trypsin-EDTA-solution. The cells were further passaged to three wells of a 6-well plate. These cells were either used fresh after being passaged to 6 wells of a 6-well plate or were cryopreserved in cryo-serum free medium (Cryo-SFM) (Gibco, Life Technologies, Paisley, UK,) and stored at −80 °C until further use.

### XTT assay

Cell viability was evaluated using the XTT assay and was performed along the manufacturer`s instructions. pLECs were seeded into 96-well cell culture plates in KSFM. At 70–80% confluence, the culture medium was changed to a culture medium containing drugs (duloxetine/ritanserin) for 24 h and then XTT solution was prepared fresh and added to each well. As a negative control, XTT solution was added to a well with cells but without duloxetine and ritanserin. Culture well plates were incubated for about 30–60 min and absorbance were measured at 550 nm on a 96-well microplate reader (TECAN Infinite F50).

### siRNA transfection of pLECs

Either the cryopreserved pLECs were thawed at 37°C for a few seconds and were seeded into 6-well plates or freshly isolated and cultured cells without cryopreservation were used in the 6-well plates. After the cells reached 70–80% confluence, transfection was performed, as previously described [34]. For cells in each well of a 6-well plate, 5 nM of both canonical PAX6 and PAX6 5a isoforms (5′CCUGGCUAGCGAAAAGCAAUU and 5′UGGGCGGAGUUAUGAUACCUU) was used in combination (si-PAX6 pLECs) for PAX6 knockdown. As a control, 5 nM of non-specific control siRNA (5′AGGUAGUGUAAUCGCCUUGUU) (si-CTRL pLECs) was used. Cells were transfected using 5 μL of Lipofectamine 2000 reagent (1 mg/mL; Invitrogen, CA, USA) diluted in 150 μL of Opti-MEM + GlutaMAX-I (Gibco, Carlsbad, CA, USA) per well. Transfection was carried out for 48 hours at 37 °C.

In addition, in order to reduce PAX6 knockdown level, 2.5 nM of both canonical PAX6 and PAX6 5a isoforms in combination (si-PAX6) were also used, in separate experiments. For these experiments, 2.5 nM of non-specific control siRNA (si-CTRL) served as the transfection control. The incubation time for these transfections was 48 hours at 37 °C.

The typical cobblestone morphology of untransfected, si-CTRL and si-PAX6 pLECs is shown in S1 Fig. Cell morphology remained unchanged across the different transfection conditions.

### Duloxetine and Ritanserin treatment

Duloxetine (SML0474, Sigma-Aldrich, Massachusetts, USA) and Ritanserin (R-103 Sigma-Aldrich, Massachusetts, USA) were dissolved in Dimethyl sulphoxide solution (DMSO) to prepare 1 mM stock solution. First, the transfection reagent was removed from the cell cultures (after 48 hours), and the cells were washed with PBS. The transfected cells were then incubated in KSFM without epidermal growth factor (EGF) (starvation medium) for 24 hours. For drug treatment, the transfected cells were first incubated with 5 ng/ml EGF-containing KSFM and kept in the incubator for 30 minutes before adding the drugs. PAX6 siRNA-transfected cells were divided into three groups. One group was incubated with 5 µM DMSO in KSFM with EGF, a second group was incubated with 5 µM duloxetine in KSFM with EGF, and a third group was incubated with 5 µM ritanserin in KSFM with EGF for 24 hours at 37 °C. Additionally, control siRNA-transfected cells were also incubated with 5 µM DMSO.

Additionally, both primary control and primary aniridia cell cultures were divided into three groups, and each group was either treated with DMSO, 5 µM duloxetine or 5 µM ritanserin, for 24 h at 37 °C, respectively.

### RNA isolation and quality control

RNA/protein was extracted using RNA/DNA/Protein Purification Plus Kit (Norgen, Thorold, ON, Canada, cat no. 47700), according to the manufacturer’s instruction manual. After the drug treatment, cells were washed with PBS and lysed by adding 300 µl guanidinium salts containing SKP-lysis buffer (3 µl-betamercaptoethanol was added) (RNA/DNA/Protein Purification Plus Kit, Norgen Biotek, Canada) for 5 min at room temperature. To remove genomic DNA, the cell lysates were loaded onto a gDNA purification column and were centrifuged at 5200g for 2 min. The flowthrough from the column was retained for RNA purification, as it contains RNAs, and proteins. RNA was precipitated by adding 60 µl 96–100% ethanol to every 100 µl flowthrough and was loaded onto the RNA/Protein Purification Column and was centrifuged at 3500g for 2 min. Protein purification was also performed from the RNA flowthrough therefore retained for later and the column was washed three times with wash solution for RNA elution. Thereafter, total RNAs were eluted in 30 µl RNA Elution Buffer by centrifugation at 200g for 2 min. RNA concentration was quantified using Nanodrop 2000 spectrophotometer (Thermo Fisher Scientific, Waltham, MA, USA) for the quality control. The pH of the RNA flowthrough was adjusted by adding 100 µl molecular biology grade water and 8 µl binding buffer to every 100 µl flowthrough. This pH adjusted sample was loaded onto the protein column and was centrifuged at 5200g for 1 min. The column was washed twice with protein wash solution. Finally, protein was eluted with 50 µl protein elution buffer and 9.3 µl protein neutralizer by centrifugation at 5200g for 2 min. Bradford assay using Bradford reagent (Sigma-Aldrich GmbH, Deisenheim, Germany) was used to determine protein concentration of the eluted protein samples.

### Reverse transcription quantitative real time PCR (RT-qPCR) to determine gene expression

cDNA synthesis was carried out using 500 ng-1 µg RNA with oligo dt primers, M-Mul V reaction buffer and Enzyme Mix (One Taq RT-PCR kit, NEB) using PCR Thermocycler (Applied bioscience), according to the manufacture`s instruction manual in a 20 µl of total reaction. For qPCR, in 10 µl total reaction, 1 µl of diluted cDNA (30 µl water added to 20 µl cDNA) was used to run the reaction. The qPCR was performed in a 96 well plate using ACEq DNA SYBR Green master Mix (Vazyme, Biotech, Nanjing). The reaction was performed in duplicate, using a PCR Thermocycler (Quant studio 5, Applied Biosystems, Waltham, Massachusetts, USA). Glucuronidase beta (GUSB), TATA-box binding protein (TBP), Beta-Actin (ACTB) and Glycerinaldehyd-3-phosphat-Dehydrogenase (GAPDH) were used as reference genes and were run under the same conditions as the target genes. The amplification conditions for the reaction mix were 95 °C for 2 min PCR initial denaturation, 95°C for 10s denaturation, and 60°C for 30s combined annealing/extension. These PCR conditions were repeated for 40 cycles. The splice variants PCR reactions for PAX6 isoforms were carried out using 2µl of cDNA, 500nM of each primer, 200nM probe and TaqMan Fast Advanced MasterMix (applied biosystems). The thermal cycling conditions were initial denaturation for 95°C for 10 min, 95°C for 15 s and 60°C for 1 min and repeated for 40 cycles. GUSB and TBP was used as a reference gene The gene expression level was analysed with QuantStudio™ (Applied Biosystems, Waltham, Massachusetts, USA) design and analysis software. Primers used for target genes and splice variants are displayed in Tables 3 and 4 respectively. The relative expression of each target gene was normalized to the reference gene and ΔΔCt values and expression fold-changes (2 ^**ΔΔCt**^ values) were calculated.

**Table 3:** Primer pairs used for qPCR.

Primers	Source
***ABCG2*:** 114 bp (NM_004827)	QT00073206
***ADH7*:** 85 bp (NM_000673, NM_001166504)	QT00000217
***ALDH1A1*:** 97 bp (NM_000689)	QT00013286
***DSG1*:** 96 bp (NM_001942)	QT00001617
***FABP5*:** 97 bp (NM_001444)	QT00225561
***GUSB*:** 96 bp (NM_000181, NM_001284290, NM_001293104,	QT00046046
NM_001293105)	
***KRT3*:** 118 bp (NM_057088)	QT00050365
***KRT12*:** 104 bp (NM_000223)	QT00011949104
***PAX6*:** 113 bp (NM_000280, NM_001127612, NM_001604,	QT00071169
NM_001258462, NM_001258463, NM_001258464,	
NM_001258465)	
***TBP*:** 132 bp (NM_001172085, NM_003194)	QT00000721
***SRY:*** 131 bp (NM_003140)	QT00199913
***NLGN4X/Y:*** 381 bp(X), 187 bp(Y)	Maxeiner et al. [35]

**Table 4:** Primers used for TaqMAN assay qPCR.

Primer	Sequence	Source
PAX6 forward	GGCCGTGCGACATTTCC (17 bp)	Eurofins scientific
PAX6 reverse	ACCTGCCCAGAATTTTACTCACA (23 bp)	Eurofins scientific
PAX6−5 isoform probe FAM BHQ MGB	AATTCTGCAGGTGTCCAA (18 bp)	Eurofins scientific
PAX6-5a isoform probe VIC/YAKIMA BHQ MGB	CCCATGCAGATGCAA (15 bp)	Eurofins scientific
TBP FAM MGB	91 bp	Thermo Fischer Hs00427620_m1/ 4331182
GUSB FAM MGB	96 bp	Thermo Fischer Hs00939627_m1/ 4453320

### Western blot

Nu Page™ Bis-Tris SDS Gel (4–12%) (Invitrogen, Waltham, MA, USA) was used to perform the western blot. 10 μg of each control siRNA and PAX6 siRNA protein sample (with or without previous drug treatment) was boiled in Laemmli sample buffer for 5 min at 95 °C. Denatured samples and Dual colour marker (Bio-Rad Laboratories, Munich, Germany) were loaded and run at 100 V for 2 h. Semi dry blot system was used to transfer the separated high molecular weight proteins to nitrocellulose membrane, using Trans-blot turbo transfer pack (Bio-Rad, Herculus CA, USA) with the Trans-Blot Turbo Transfer System (Bio-Rad, Herculus CA, USA) for 10 min. The membrane was washed with water and subjected to No-stain protein labelling reagent (Invitrogen CA, USA) for total protein staining. Primary antibodies were diluted according to the concentrations listed in Table 5, using the Western Foxx Kit solution (Bio Froxx GmbH, Einhausen, Germany), which contains both the blocking buffer and secondary antibody solution. Western lightning chemiluminescence reagent plus ECL (Perkin Elmer Life Science) was used for the band detection. Images were acquired with an iBright 1500 system (Invitrogen CA, USA). The quantification analysis of band signal intensity of each antibody was done by normalization with total protein staining (TPN) of western blots [38] (S2 Fig.). The antibodies used for the western blot analysis are listed in Table 5. Densitometric analysis of western blots was performed using iBright analysis software (Invitrogen CA, USA).

**Table 5:** Antibodies used for western blot.

Primary antibodies	Source	Dilutions (WB)
PAX6	Sc-32766, Santa Cruz Biotechnology, California, USA	1:1000
pERK (Phospho-p44/42 MAPK (Thr202/Tyr204)	#4370, Cell signalling technology, Cambridge, UK	1:1000
ERK (p44/42 MAPK (Erk1/2) (137F5)	#4695, Cell signalling technology, Cambridge, UK	1:1000

### Statistical analysis

Graph Pad Prism 7.04 software (CA, USA) was used for analysis and for drawing the graphs. mRNA and protein expression values were exported as excel files. Fold change and TPN normalized western blot signal intensity were analysed using Shapiro wilk normality test. Data values were normally distributed therefore, one-way ANOVA, followed by Dunnet’s test was used. A p-value below 0.05 was considered statistically significant.

## Results

### Effect of duloxetine and ritanserin on the primary siRNA based aniridia cell model

Duloxetine and ritanserin drugs were selected from FDA approved and bioactive drug library (Microsource Spectrum Collection). The screening was done for both drugs using TRE-tomato-HEK293 cells that have a *PAX6*-responsive sequence upstream to tomato reporter [39,40]. Thus, the purpose of the study was to test the effect of these drugs on PAX6.

**Fig 1:**
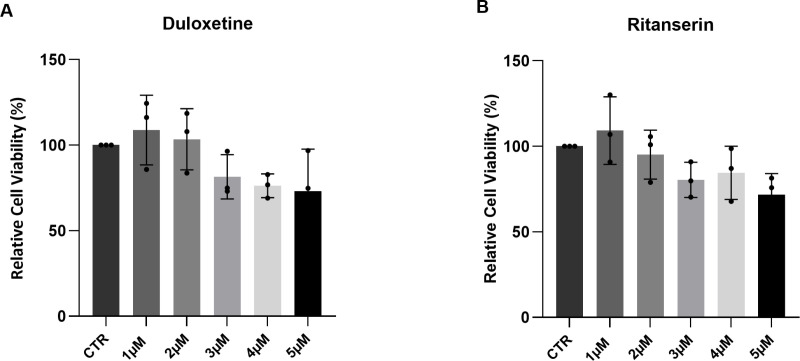
Cell viability assay (A, B). The survival of primary limbal epithelial cells (pLECs) was assessed following 24-hour treatment with duloxetine (1–5 µM, A) and ritanserin (1–5 µM, B) using the XTT assay. Neither duloxetine nor ritanserin significantly affected pLEC viability (p ≥ 0.08). Data are presented as mean ± SD (one-way ANOVA, n = 3). Abbreviation: CTR, healthy control pLECs.

Duloxetine and ritanserin were tested on primary limbal epithelial cells (pLECs) in which PAX6 gene was knocked down using a siRNA against *PAX6* in pLECs. Since aniridia is primarily associated with *PAX6* haploinsufficiency, therefore, siRNA PAX6 knockdown can mimic aniridia *PAX6* deficiency in pLECs. To estimate the appropriate drug concentration, which was to be used for the subsequent treatments, the cell viability assay was performed. Cell viability assay (XTT) determined the pLECs survival following duloxetine and ritanserin treatment for 24 h. Nevertheless, no significant cell viability change was observed after treatment of pLECs with increasing concentrations (1 µM to 5 µM) of duloxetine (p ≥ 0.01, n = 3) (Fig 1A) and ritanserin (p ≥ 0.08, n = 3) (Fig 1B).

Initially, we tested 1 µM concentrations of both compounds, as this dose had minimal impact on cell viability. However, at this lower concentration, no significant change in *PAX6* mRNA expression was observed following treatment (S3 Fig.). 5 µM duloxetine and ritanserin were well tolerated through pLECs, therefore, these concentrations were used for the subsequent treatment of pLECs.

**Fig 2:**
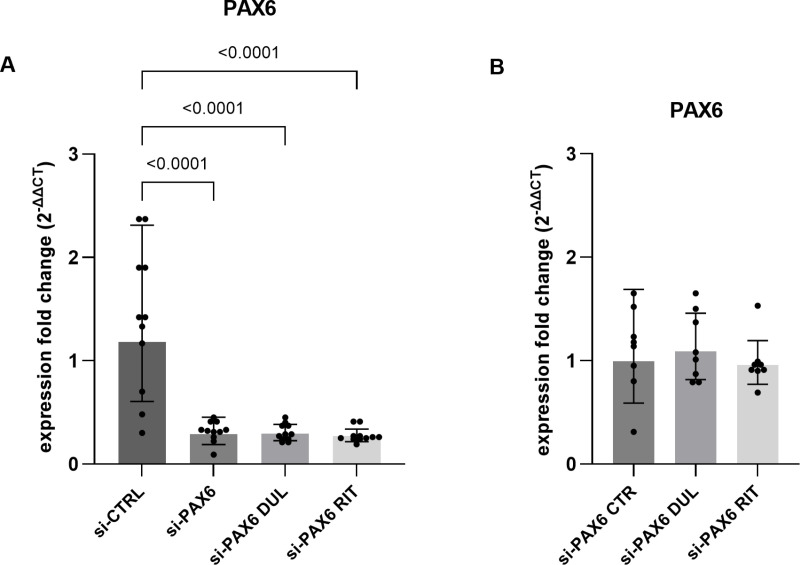
*PAX6* mRNA level in primary limbal epithelial cells (pLECs) transfected with 5 nM siRNA (A, B). (A) Quantitative RT-PCR analysis of *PAX6* expression in pLECs transfected with 5 nM PAX6 siRNA and 5 nM non-specific control siRNA (si-CTR) for 48 h. The fold change (FC) in expression relative to the control (si-CTR) confirms successful knockdown of PAX6 (p < 0.0001). (B) Fold changes in PAX6 expression in PAX6 siRNA-transfected pLECs treated with 5 µM duloxetine (si-P*AX6* DUL) or 5 µM ritanserin (si-*PAX6* RIT) for 24 h, compared to PAX6 siRNA-transfected cells without drug treatment (si-*PAX6* CTR). Drug treatment had no significant impact on PAX6 mRNA expression (p ≥ 0.8). Data are represented as geometric mean ± geometric SD (one-way ANOVA, n = 8).

Treatment with 5 µM duloxetine for 24 h had no significant effect on *PAX6* mRNA expression in pLECs transfected with 5 nM siRNA against PAX6, compared to untreated transfected controls (si-PAX6 CTR), as determined by qRT-PCR (p = 0.9, n = 8; Fig 2B). Similarly, treatment with 5 µM ritanserin for 24 h did not significantly influence *PAX6* mRNA expression (p = 0.7, n = 8; Fig 2B).

**Fig 3:**
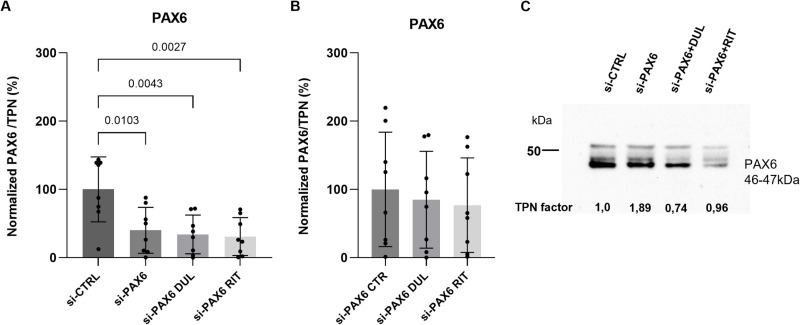
PAX6 protein level analysis using Western blot (A-C). (A) Western blot analysis showing PAX6 protein expression in primary limbal epithelial cells (pLECs) transfected with 5 nM PAX6 siRNA, compared to cells transfected with 5 nM non-targeting control siRNA (si-CTRL), after 48 h. A significant reduction in PAX6 protein levels was observed in the knockdown group (p ≤ 0.01). (B) PAX6 protein expression in PAX6-siRNA-transfected pLECs treated with 5 µM duloxetine (si-PAX6 DUL) or 5 µM ritanserin (si-PAX6 RIT) for 24 h, compared to untreated PAX6 siRNA-transfected cells (si-PAX6 CTR). Neither drug significantly enhanced PAX6 protein expression (p = 0.8). (C) Representative Western blot image showing PAX6 protein bands at the expected molecular weight of 46–47 kDa. Data are presented as mean ± SD (one-way ANOVA, n = 8).

In contrast, a significant reduction in *PAX6* mRNA levels was observed in siRNA-PAX6-transfected pLECs compared to cells transfected with non-specific control siRNA (si-CTRL), confirming successful knockdown (p < 0.0001, n = 8; Fig 2A). Light microscopic evaluation showed no noticeable changes in pLEC morphology after transfection, indicating that the transfection reagents did not affect cell viability (S1 Fig.). At the protein level, neither duloxetine nor ritanserin treatment enhanced PAX6 expression (p ≥ 0.8), following the significant reduction observed after PAX6 knockdown (p ≤ 0.01, n = 8; Fig 3A–3C).

**Fig 4:**
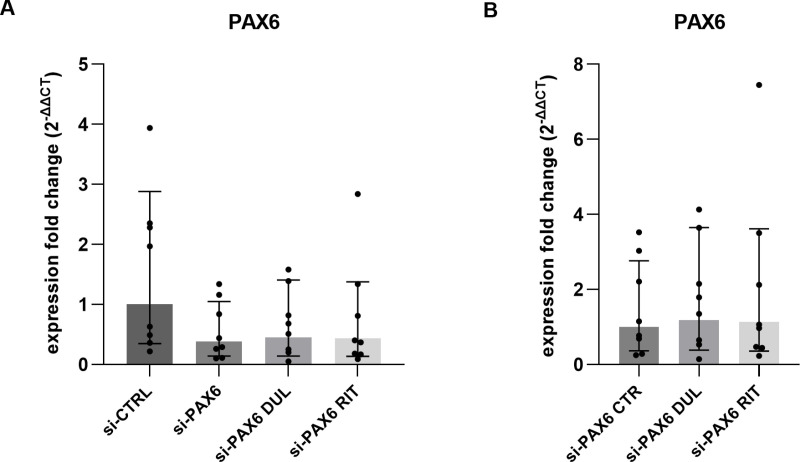
*PAX6* mRNA level in primary limbal epithelial cells (pLECs) transfected with 2.5 nM siRNA (A, B). (A) Quantitative RT-PCR analysis of *PAX6* mRNA expression in pLECs transfected with 2.5 nM PAX6 siRNA and 2.5 nM control siRNA for 48 h. Expression fold changes (FC) relative to a non-specific siRNA control (si-CTRL) are also shown. (B) Expression fold changes in transfected pLECs treated with 5 µM duloxetine (si-PAX6 DUL) or 5 µM ritanserin (si-PAX6 RIT) for 24 h, compared to PAX6 siRNA-transfected cells without drug treatment (si-PAX6 CTR). Treatment with duloxetine (p = 0.9) or ritanserin (p = 0.8) did not significantly affect *PAX6* mRNA expression. Data are presented as geometric mean ± geometric SD (one-way ANOVA, n = 8).

To rule out the possibility that the lack of drug effect on PAX6 expression was due to a higher concentration of transient siRNA in pLEC cultures, transfections were also performed using a lower concentration (2.5 nM) of PAX6 siRNA and non-specific control siRNA (si-CTRL) (p ≥ 0.1). Nevertheless, treatment with 5 µM duloxetine or ritanserin did not significantly affect PAX6 mRNA expression compared to the control without drug treatment (si-PAX6 CTR) (p ≥ 0.8, n = 8) (Fig 4A, 4B).

**Fig 5:**
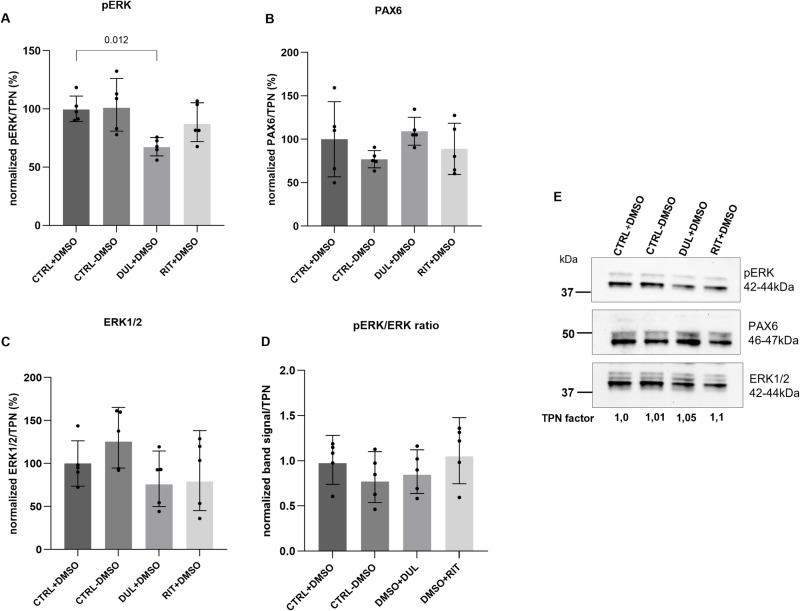
pERK and PAX6 protein level analysis in primary limbal epithelial cells (pLECs) using Western blot (A-E). Western blot analysis of pERK and PAX6 protein expression in untransfected pLECs, with and without DMSO treatment (CTRL+DMSO; CTRL-DMSO), and after treatment with duloxetine (DUL+DMSO) or ritanserin (RIT+DMSO) (A-E). Western blot images show pERK/ERK1/2 bands at 42–44 kDa and PAX6 bands at 46–47 kDa. (A) 5 µM duloxetine treatment led to a significant reduction in pERK1/2 protein expression compared to controls (p = 0.012). (B) Duloxetine treatment did not significantly increase PAX6 protein expression. (C) The pERK/total ERK ratio remained unchanged between the groups. Data are presented as mean ± SD (one-way ANOVA, n = 5).

These findings were surprising as duloxetine and ritanserin enhanced endogenous PAX6 expression in mut-limbal stem cells LSCs. The potential mechanism of action of duloxetine and ritanserin in LSCs is the activation of PAX6 by inhibiting the mitogen-activated protein kinase (ERK) pathway [39,40] Therefore, we investigated whether the duloxetine and ritanserin treatment influence phosphorylated ERK (pERK1/2) protein expression in untransfected pLECs. We observed that 5µM duloxetine treatment led to significant reduction of the pERK1/2 protein expression, compared to controls (using only DMSO) (p = 0.01, n = 5) (Fig 5A, 5E). Nevertheless, duloxetine treatment did not significantly increase the PAX6 protein expression (p = 0.9) (Fig. 5B, 5E). Ritanserin treatment had no significant effect on pERK expression (p = 0.6) and had no effect on PAX6 protein expression (p = 0.8) as well (Fig 5A-5E). The total ERK1\2 was unchanged between the groups (p ≥ 0.4) (Fig 5 C, 5E). The expression level of pERK relative to total ERK in duloxetine- and ritanserin-treated pLECs was also not significantly different from the control group (p ≥ 0.5; Fig 5D).

### Effect of duloxetine and ritanserin treatment on PAX6 isoforms in primary cell cultures, in vitro

**Fig 6:**
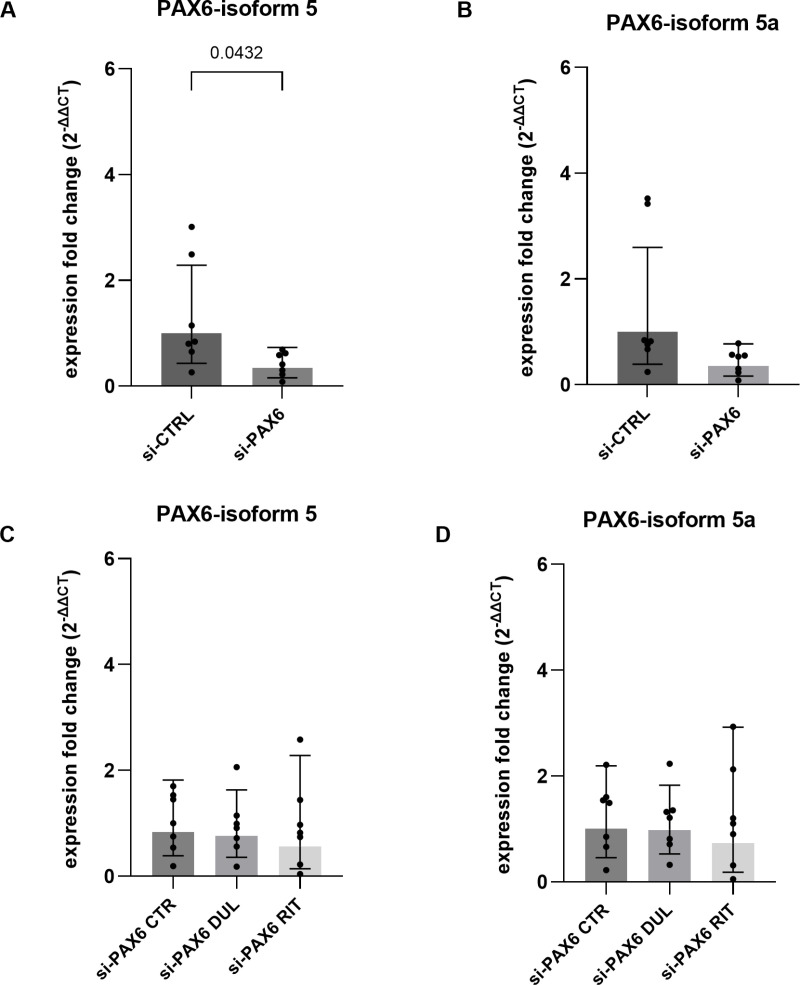
*PAX6* isoform 5 and 5a mRNA level measurement (A-D). TaqMan qRT-PCR analysis of PAX6 isoform expression in primary limbal epithelial cells (pLECs) transfected with PAX6 siRNA (si-PAX6) or control siRNA (si-CTRL) for 48 h, followed by treatment with 5 µM duloxetine (si-PAX6 DUL) (C) or 5 µM ritanserin (si-PAX6 RIT) (D) for 24 h. Expression fold changes (FC) for PAX6 isoform 5 (A, C) and PAX6 isoform 5a (B, D) are shown. (A, B) PAX6−5 isoform mRNA levels were significantly reduced following siRNA-mediated knockdown (p = 0.043), whereas the reduction in PAX6-5a isoform mRNA levels did not reach statistical significance (p = 0.07). (C, D) Treatment with duloxetine and ritanserin did not significantly alter the expression of either isoform (p ≥ 0.8). Data are presented as geometric mean ± geometric SD (one-way ANOVA, n = 7).

The PAX6 has two major isoforms, the canonical *PAX6−5* and the *PAX6-5a* isoforms. *PAX6-5a* is an alternatively spliced form that results in a larger isoform than *PAX6−5*. Previous studies have shown that these two PAX6 isoforms regulate corneal epithelium specific genes differentially as well as cooperatively [41,42]. Therefore, the effect of duloxetine and ritanserin treatment was examined on these both isoforms, to evaluate whether these drugs have a different effect on both isoforms. The *PAX6* isoform-5 mRNA levels were significantly reduced after siRNA PAX6 knockdown (p = 0.04). However, the change in mRNA levels of the PAX6-5a isoform was not statistically significant (p = 0.07) (Fig 6A, 6B). Unlike previous findings, although a relative increase in PAX6 mRNA levels was observed after duloxetine and ritanserin treatment, the expression levels of both PAX6 isoforms remained unchanged and were not statistically significant (p ≥ 0.1, n = 8) (Fig 6C, 6D).

### Effect of duloxetine and ritanserin treatment on aniridia patient`s derived primary limbal epithelial cell cultures, in vitro

**Fig 7:**
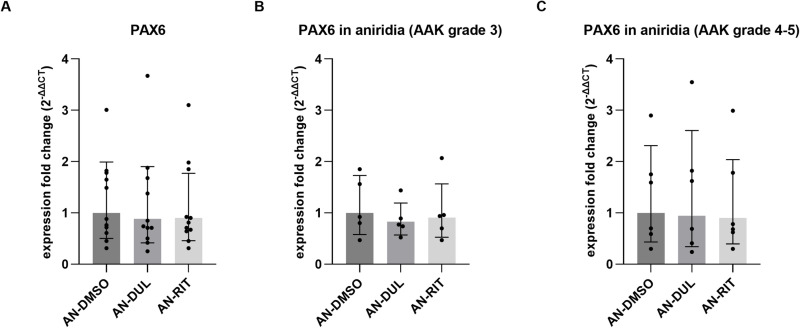
Impact of duloxetine and ritanserin on aniridia patient-derived primary limbal epithelial cells (AN-pLECs)(A-C). (A) qRT-PCR analysis of *PAX6* mRNA expression in AN-pLECs treated with 5 µM duloxetine or 5 µM ritanserin for 24 h. Expression fold changes (FC) are shown relative to untreated controls. (B) PAX6 expression in AN-pLECs derived from patients with AAK Grade 3, following 24 h treatment with duloxetine or ritanserin. (C) PAX6 expression in AN-pLECs from patients with AAK Grades 4–5 under the same treatment conditions. No significant changes in *PAX6* mRNA expression were observed in any group following treatment with duloxetine or ritanserin. Data are presented as geometric mean ± geometric SD (one-way ANOVA, n = 11). Abbreviation: AN, aniridia.

Since we did not observe significant influence of duloxetine and ritanserin on siRNA-PAX6 transfected pLECs, thus, we investigated the impact of duloxetine and ritanserin treatment on aniridia patient derived primary limbal epithelial cells (AN-pLECs). Unfortunately, cell viability could not be performed due to low availability of primary aniridia limbal epithelial cells. Therefore, the effect of 5 µM duloxetine and ritanserin treatment was tested on AN-pLECs for 24 h based on the cell viability assay performed on healthy donor pLECs. We could observe similar results, as using the siRNA PAX6 knockdown133 pt for pLEC previously, as 5µM duloxetine and 5µM ritanserin treatment had no significant effect on *PAX6* mRNA expression in AN-pLECs, as judged by qPCR analysis (p = 0.9, n = 11) (Fig 7A). The aniridia samples included in this study exhibited varying AAK grades (Table 1). To account for this, the samples were divided into two groups: Group 1 included samples with AAK Grade 3, while Group 2 comprised samples with AAK Grades 4 and 5. Treatment with 5 µM duloxetine or 5 µM ritanserin had no significant effect on PAX6 mRNA expression in either the AAK Grade 3 group (p ≥ 0.7, n = 5) (Fig 7B) or the AAK Grade 4–5 group (p = 0.9, n = 6) (Fig 7C).

### Target gene expression after duloxetine and ritanserin treatment

**Fig 8:**
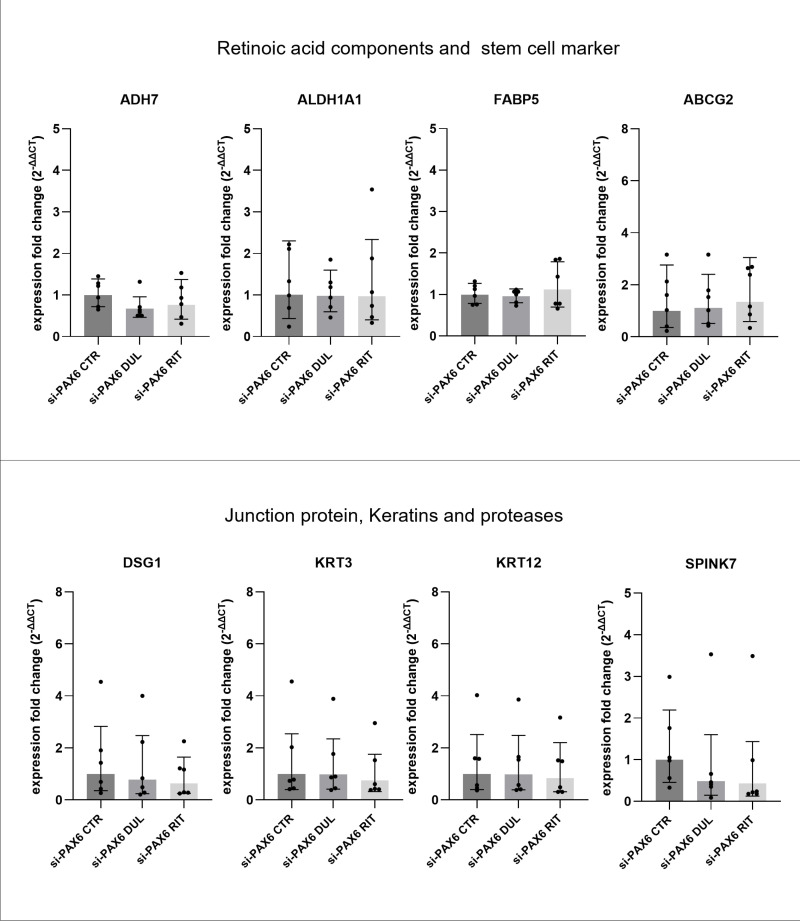
mRNA level analysis of *PAX6* target genes. qRT-PCR analysis of selected PAX6 target genes in transfected primary limbal epithelial cells (pLECs) treated with 5 µM duloxetine (si-PAX6 DUL) or 5 µM ritanserin (si-PAX6 RIT) for 24 h, using the siRNA-based PAX6 knockdown model (si-PAX6). Expression fold changes (FC) are shown relative to the PAX6 siRNA control without drug treatment (si-PAX6 CTR). None of the analyzed genes showed significant changes in expression following duloxetine or ritanserin treatment. Data are presented as geometric mean ± geometric SD (one-way ANOVA, n = 6).

The components of retinol and fatty acid metabolism (ADH7, ALDH1A1, FABP5), Junction protein DSG1, keratins (KRT3, KRT12) and the protease inhibitor SPINK7 were already known to be altered in aniridia patient epithelial cells and using the siRNA-based aniridia cell model [34,36,43]. Therefore, we analysed the expression changes of these genes in response to duloxetine and ritanserin treatment. 5µM duloxetine and ritanserin treatment for 24 h showed no significant impact on *ADH7, ALDH1A1, FABP5* mRNA expression (p ≥ 0.2) (Fig 8A). In addition, *KRT3* and *KRT12* mRNA expression and *DSG1* and *SPINK7* mRNA expression also did not change significantly upon drug treatment (p ≥ 0.6) (Fig 8B). We also assessed gene expression of the stem cell marker *ABCG2*, which was upregulated in AN-pLECs and is negatively correlated with *ADH7* expression [34]. Nevertheless, the *ABCG2* transcript level also remained unchanged after duloxetine and ritanserin treatment (p = 0.8) (Fig 8A).

## Discussion

In this study, we evaluated the impact of duloxetine and ritanserin drugs on primary limbal epithelial cells in in vitro cultures, using the siRNA based aniridia PAX6 knockdown model and using primary cells derived from biopsies of healthy and aniridia patients. Aniridia is a rare disease and is particularly characterized by ocular surface disease known as aniridia associated keratopathy (AAK), which is progressive in nature. AAK is often associated with conjuctivalization and limbal stem cell deficiency, and thereby with loss of corneal limbal epithelial stem cell function [1–5]. In almost 90% of aniridia cases there is PAX6 haploinsufficiency and most of the patients with PAX6 mutation develop AAK. Thus, there is a current need to identify therapeutic options to prevent corneal opacification and visual loss in congenital aniridia subjects. Since AAK is progressive in nature, therefore, identifying new therapeutic options have great potential to arrest the progression of disease at early stages.

In a previous study, Roux et al., 2018 screened the FDA-approved drug library and identified two psychotropic drugs, duloxetine and ritanserin, which enhanced expression of PAX6 in mut LSCs. The mut LSCs were immortalized PAX6^+/−^ limbal epithelial cells, generated by CRISPR-CAS technology [25,39,40]. The drug “repurposing” or drug “reprofiling” is a strategy to use approved or investigational drugs for a new purpose. This strategy is quite advantageous over developing an entirely new drug, as in case of a repurposed drug, the rate of failure is relatively low with a drug which was already found safe to be used in humans. AAK is highly related to deficiency in PAX6, however, due to the complexity of AAK pathogenesis, the observed changes might not reflect the direct influence of PAX6. Therefore, siRNA based PAX6 knockdown in primary LECs (aniridia cell model) could serve as a good model to investigate the influence of duloxetine and ritanserin on PAX6 expression [34]. The siRNA based PAX6 knockdown cell model mimics PAX6 deficiency in primary aniridia limbal epithelial cell cultures. This was already demonstrated by investigating AAK associated target genes such as *SPINK7, ADH7* and *ALDH1A1* that showed similar dysregulation in the cell model, as in primary aniridia LECs, providing evidence, that this model is useful to investigate the direct or indirect influence of PAX6 in AAK [34]. Therefore, the direct influence of duloxetine and ritanserin could be examined in the aniridia PAX6 knockdown cell model. Using siRNA, the 48h transfection time of primary limbal epithelial cells was effective in a strong reduction of PAX6 expression.

Surprisingly, although PAX6 expression was effectively reduced by siRNA-mediated knockdown, treatment with duloxetine or ritanserin did not alter endogenous PAX6 levels in the knockdown model, compared to the control knockdown. PAX6 is a key transcription factor that contains two DNA-binding domains: the paired domain (PD) and the homeodomain (HD) [41,44]. The N-terminal PAI subdomain of the paired domain includes an alternatively spliced exon, exon 5a, which results in two distinct isoforms of PAX6 [45,46]. This structural variation in the PAI domain imparts unique functional properties to each isoform [42,46]. Splice variant analysis showed a significant reduction in the expression of the PAX6−5 isoform following PAX6 knockdown, compared to control knockdown. Although the siRNA used targets both PAX6 isoforms, the expression of PAX6-5a was also reduced, but the decrease did not reach statistical significance. Despite the strong downregulation of the PAX6−5 isoform, treatment with duloxetine and ritanserin did not significantly increase PAX6 expression, contrary to expectations. Since the two PAX6 isoforms are known to cooperatively regulate different target genes [41], it is possible that the relatively higher expression level of PAX6-5a in knockdown cells, compared to PAX6−5, may have compensated for the effect of duloxetine and ritanserin on PAX6 expression. Therefore, we could not recapitulate completely the effect of duloxetine and ritanserin using the siRNA based aniridia cell model, as described previously for the CRISPR-CAS modified cells [39,40]. Similar discrepancy was also observed for primary limbal epithelial cells from aniridia patients, where duloxetine and ritanserin did not enhance the PAX6 gene expression.

Aniridia-associated keratopathy typically progresses through four to five stages, classified as AAK grades 1–5. However, *PAX6* mRNA expression remained unchanged in aniridia samples from both AAK grade 3 and AAK grades 4–5. This could be due to the fact that the *PAX6* mRNA level did not differ between healthy and aniridia patient pLECs [36,43] and aniridia (AN) iPSCs-derived limbal epithelial stem cells (LESCs), as described previously. The heterogeneous nature of mutations in the aniridia samples used in this study could also partly explain the lack of PAX6 upregulation following treatment with duloxetine and ritanserin.

Duloxetine is a norepinephrine and serotonin reuptake inhibitor and ritanserin, a serotonin reuptake inhibitor has been shown to be involved in the inhibition of pERK [47–50]. Previous studies have suggested that mitogen-activated protein kinase (MEK) inhibitors can indirectly increase PAX6 expression [51–53]. Rabiee et al., have demonstrated that PD0325901, a potent MEK inhibitor increased PAX6 protein expression in mutant mouse corneal epithelial cells and in PAX6-deficient aniridia, in newborn *Pax6*-deficient mice (Pax6^Sey-Neu/+^) [51]. The study by Srivastava et al., 2023 showed that PAX6 and ERK1\2 expression levels were inversely regulated and were functioning synergistically [52]. Therefore, it suggests that MEK is responsible for phosphorylation and activation of ERK and MEK inhibitors can inhibit the ERK pathway. Therefore, ERK pathway has negative feedback on PAX6 expression*.*

The transient activation of the MEK/ERK pathway, by treating the mut LSCs with EGF, reduced the PAX6 production [54,55]. Partial inhibition of ERK phosphorylation by duloxetine and ritanserin treatment has been shown to restore PAX6 expression in mutant limbal stem cells (mut LSCs) [39,40]. We observed inhibition of pERK in pLECs following treatment with duloxetine, as indicated by a reduction in pERK protein expression. In contrast, ritanserin-treated pLECs showed no evidence of pERK inhibition. The pERK level relative to total ERK also remained unchanged. Importantly, endogenous PAX6 protein expression was not enhanced in pLECs following treatment with either duloxetine or ritanserin. Interestingly, Dorot et al. (2023) reported that in wild-type limbal stem cells (LSCs), the effect of duloxetine on phosphorylated ERK was weaker compared to mutant LSCs, and endogenous PAX6 expression was not enhanced [39]. This may partially explain the absence of PAX6 rescue in our siRNA-mediated knockdown model, as these pLECs do not harbor mutations in either PAX6 allele and thus may respond differently to drug treatment.

The possibility that these differences are most likely due to the variations in the cultures systems cannot be completely ruled out. The primary LECs cultures of our group comprise a heterogenous population of cells and PAX6 expression also varies from donor to donor. The differences in the differentiation status of these cells might also contribute to the inability of duloxetine and ritanserin to enhance PAX6 expression. In contrast, the immortalized cells, used by Roux et al., 2018 express extremely low amount of markers that are related to corneal differentiation, thereby indicating their close match to stem cell phenotype [25]. In aniridia pLECs and AN iPSCs-derived LESCs, the stem cell marker ∆Np63α showed no differences in transcription level, compared to healthy controls [36,43]. Another stem cell marker, the ABCG2 mRNA level was upregulated in pLECs and iPSCs-derived LESCs from aniridia patients. The junction and keratin protein, DSG1 and KRT3 were highly significantly regulated genes that might be influenced by PAX6 expression [56–59]. The regulation of keratin protein KRT12 and RA signaling component ALDH1A1 showed overlapping results obtained from microarray of primary corneal epithelial cells [60] with aniridia patient RNA sequencing results [34]. In duloxetine and ritanserin treated pLECs, none of these genes showed significant alterations in gene expression in response to both used drugs. This could be due to the limited number of aniridia samples with very high genetic variability and different PAX6 mutations, used in our and in other studies, which makes it difficult to investigate the effects of duloxetine and ritanserin.

Such discrepancies in the siRNA based PAX6 knockdown model and in primary limbal epithelial cells from aniridia patients were also observed in qPCR experiments and using mRNA sequencing, showing a slight overlap in gene expression profiling and pathway analysis with immortalized PAX6^+/−^ limbal epithelial cells [25,34]. Nevertheless, it cannot be completely ruled out that the observed gene expression results could be observed due to differences in the methodologies or due to low availability of samples. To our knowledge, this is the first study to demonstrate the application of the repurposed drugs duloxetine and ritanserin on cultured primary aniridia limbal epithelial cells. The results suggest that it is crucial to investigate the effects of drug treatment in different culture systems to better assess the efficacy of these drugs.

The aniridia samples used in this study represent heterogeneous mutations, as obtaining patient-derived aniridia samples is extremely challenging due to their limited availability. As a result, a homogeneous genetic mutation group was not feasible and remains a limitation of our study.

In summary, duloxetine and ritanserin treatment were unable to restore endogenous *PAX6* expression in primary limbal epithelial cells with PAX6 knockdown and in primary aniridia limbal epithelial cell cultures. In this study, we observed that different culture conditions have various responses to drug treatment. Therefore, the use of in vivo models could further advance our understanding of duloxetine and ritanserin treatment in aniridia associated keratopathy.

## Supporting information

S1 Fig The typical cobblestone morphology of untransfected primary limbal epithelial cells (pLECs), as well as those transfected with 5 nM non-targeting control siRNA (si-CTRL) and 5 nM PAX6 siRNA (si-PAX6). Cell morphology remained unchanged across the different transfection conditions.
(TIF)

S2 Fig Total Protein Normalization (TPN) using Invitrogen™ No-Stain™ Protein Labeling Reagent. Following stimulation with UV light, the fluorescence of the protein labeling reagent—bound to the lysine side chains of the proteins—was detected and quantified densitometrically. TPN was used to normalize the band intensities in Western blot analysis, ensuring accurate comparison of protein expression levels across samples.
(TIF)

S3 Fig *PAX6* mRNA levels in primary limbal epithelial cells (pLECs) transfected with 5 nM siRNA and using 1 µM duloxetine or ritanserin (A, B). (A) Quantitative RT-PCR analysis of *PAX6* level in pLECs transfected with 5 nM PAX6 siRNA and 5 nM non-targeting control siRNA (si-CTRL) for 48 h. The fold change (FC) in expression relative to the control (si-CTRL) confirms successful knockdown of PAX6 (p < 0.0001). (B) Fold changes in *PAX6* expression in PAX6 siRNA-transfected pLECs treated with 1 µM duloxetine (si-P*AX6* DUL) or 1 µM ritanserin (si-PAX6 RIT) for 24 h, compared to untreated PAX6 siRNA-transfected cells (si-PAX6 CTR). Drug treatment had no significant impact on PAX6 mRNA level (p ≥ 0.8). Data are represented as geometric mean ± geometric SD (one-way ANOVA, n = 5).
(TIF)

S1 Raw_images.pdf. (PDF)
