# Supplementary figures and images for: Gene expression study in the siRNA based aniridia cell model and in primary aniridia limbal epithelial cells following duloxetine and ritanserin treatment
Source: PLoS One. 2025 Jun 10;20(6):e0324829. doi: 10.1371/journal.pone.0324829 (PMC12151445; doi:10.1371/journal.pone.0324829)

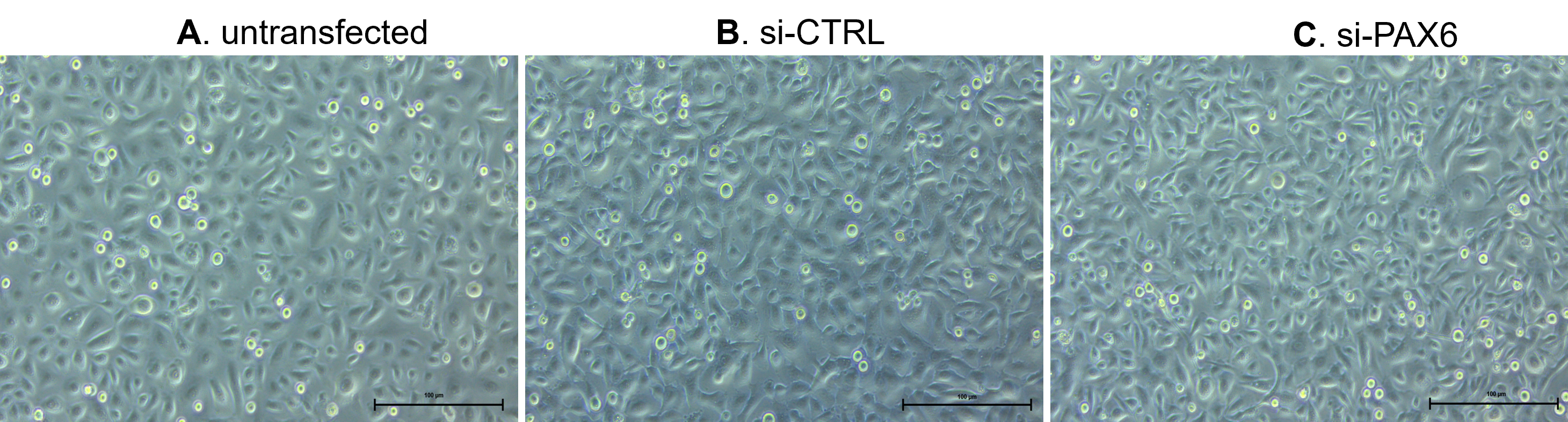

Supplement: S1 Fig — Cell morphology remained unchanged across the different transfection conditions. (TIF) [file pone.0324829.s001.tif]

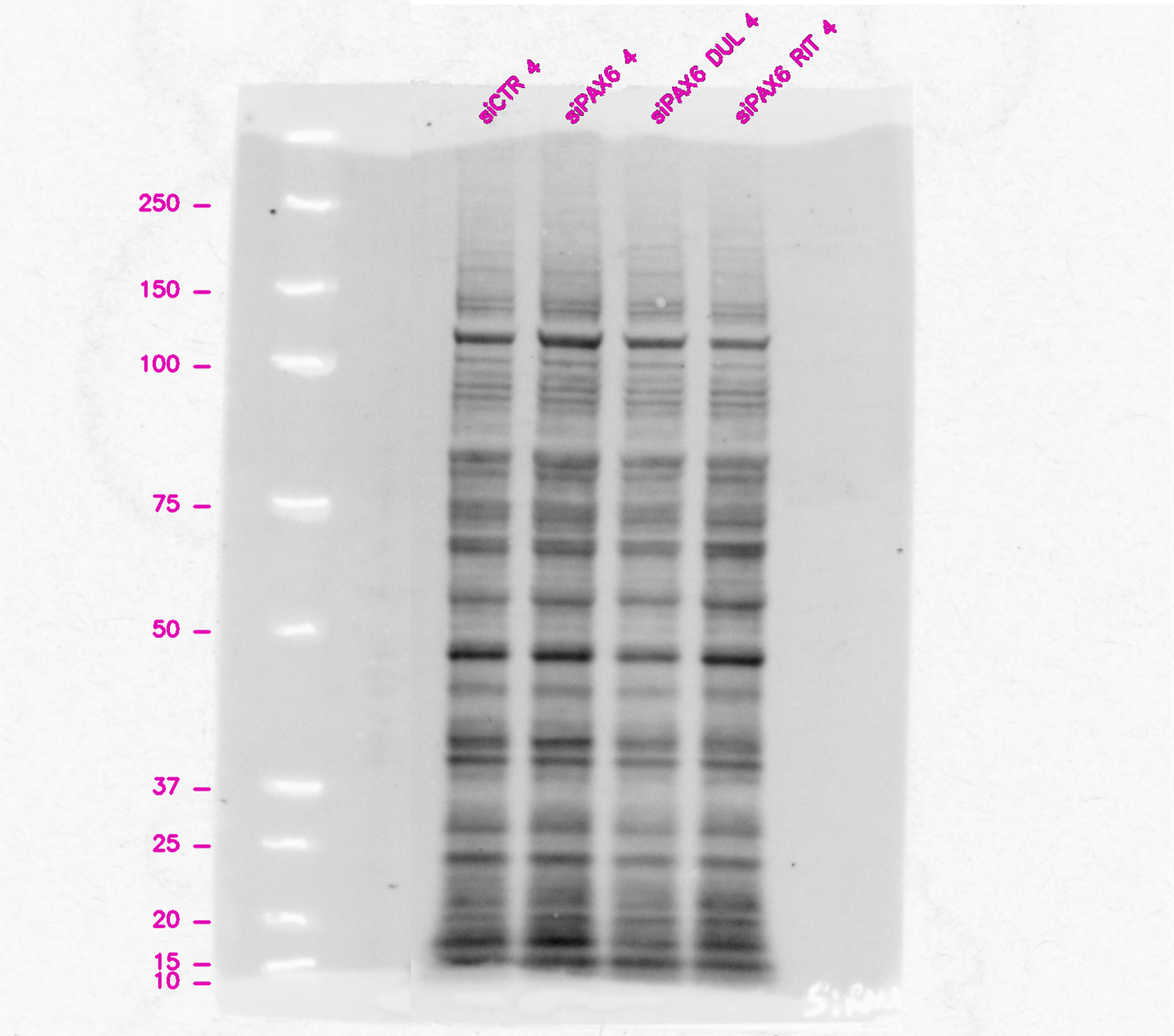

Supplement: S2 Fig — Following stimulation with UV light, the fluorescence of the protein labeling reagent—bound to the lysine side chains of the proteins—was detected and quantified densitometrically. TPN was used to normalize the band intensities in Western blot analysis, ensuring accurate comparison of protein expression levels across samples. (TIF) [file pone.0324829.s002.tif]

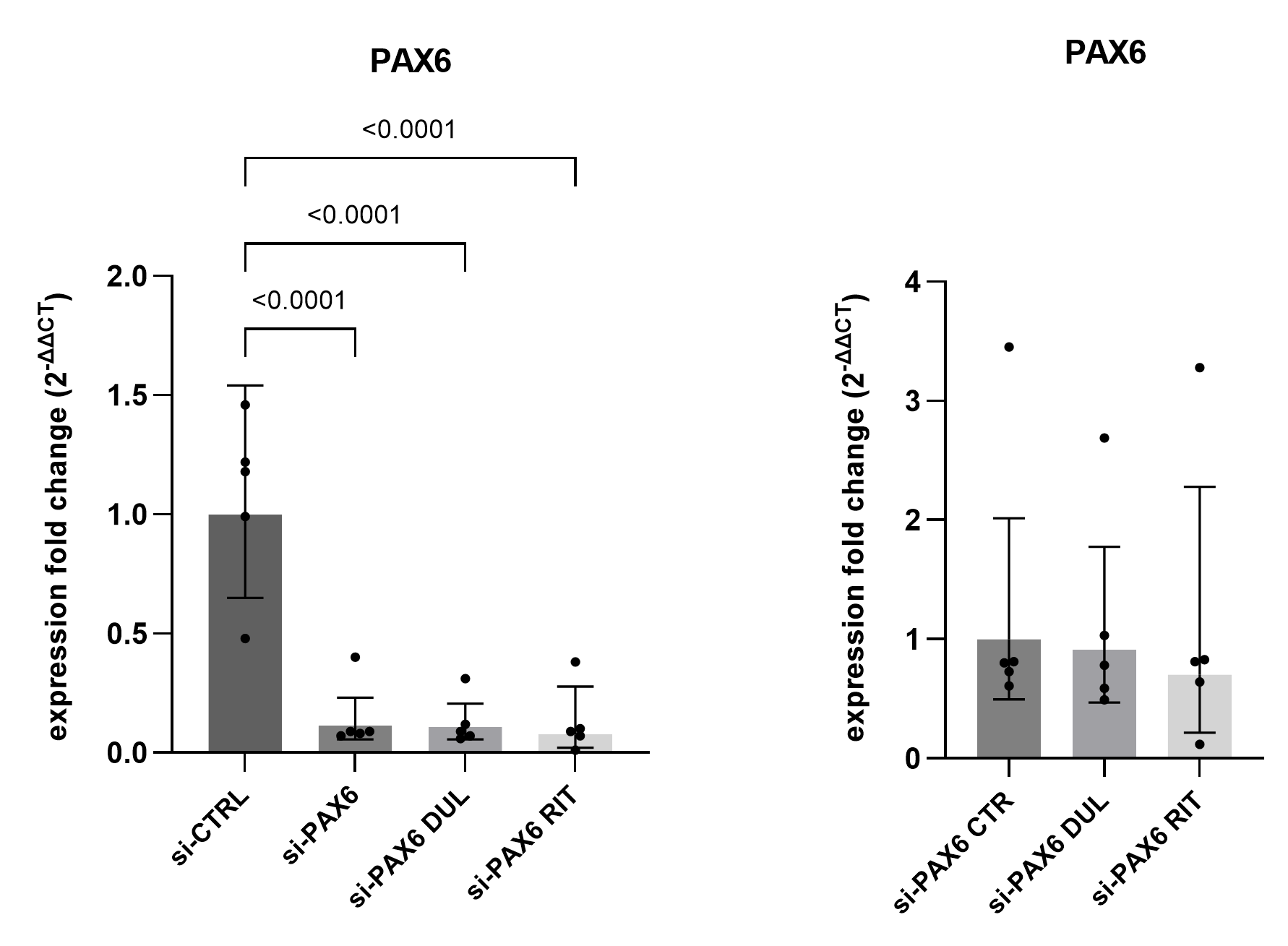

Supplement: S3 Fig — (A) Quantitative RT-PCR analysis of PAX6 level in pLECs transfected with 5 nM PAX6 siRNA and 5 nM non-targeting control siRNA (si-CTRL) for 48 h. The fold change (FC) in expression relative to the control (si-CTRL) confirms successful knockdown of PAX6 (p < 0.0001). (B) Fold changes in PAX6 expression in PAX6 siRNA-transfected pLECs treated with 1 µM duloxetine (si-PAX6 DUL) or 1 µM ritanserin (si-PAX6 RIT) for 24 h, compared to untreated PAX6 siRNA-transfected cells (si-PAX6 CTR). Drug treatment had no significant impact on PAX6 mRNA level (p ≥ 0.8). Data are represented as geometric mean ± geometric SD (one-way ANOVA, n = 5). (TIF) [file pone.0324829.s003.tif]
