# Supplementary material for: Gene expression study in the siRNA based aniridia cell model and in primary aniridia limbal epithelial cells following duloxetine and ritanserin treatment
Source: PLoS One. 2025 Jun 10;20(6):e0324829. doi: 10.1371/journal.pone.0324829 (PMC12151445; doi:10.1371/journal.pone.0324829)
Supplement: S1 — (PDF) [file pone.0324829.s004.pdf]

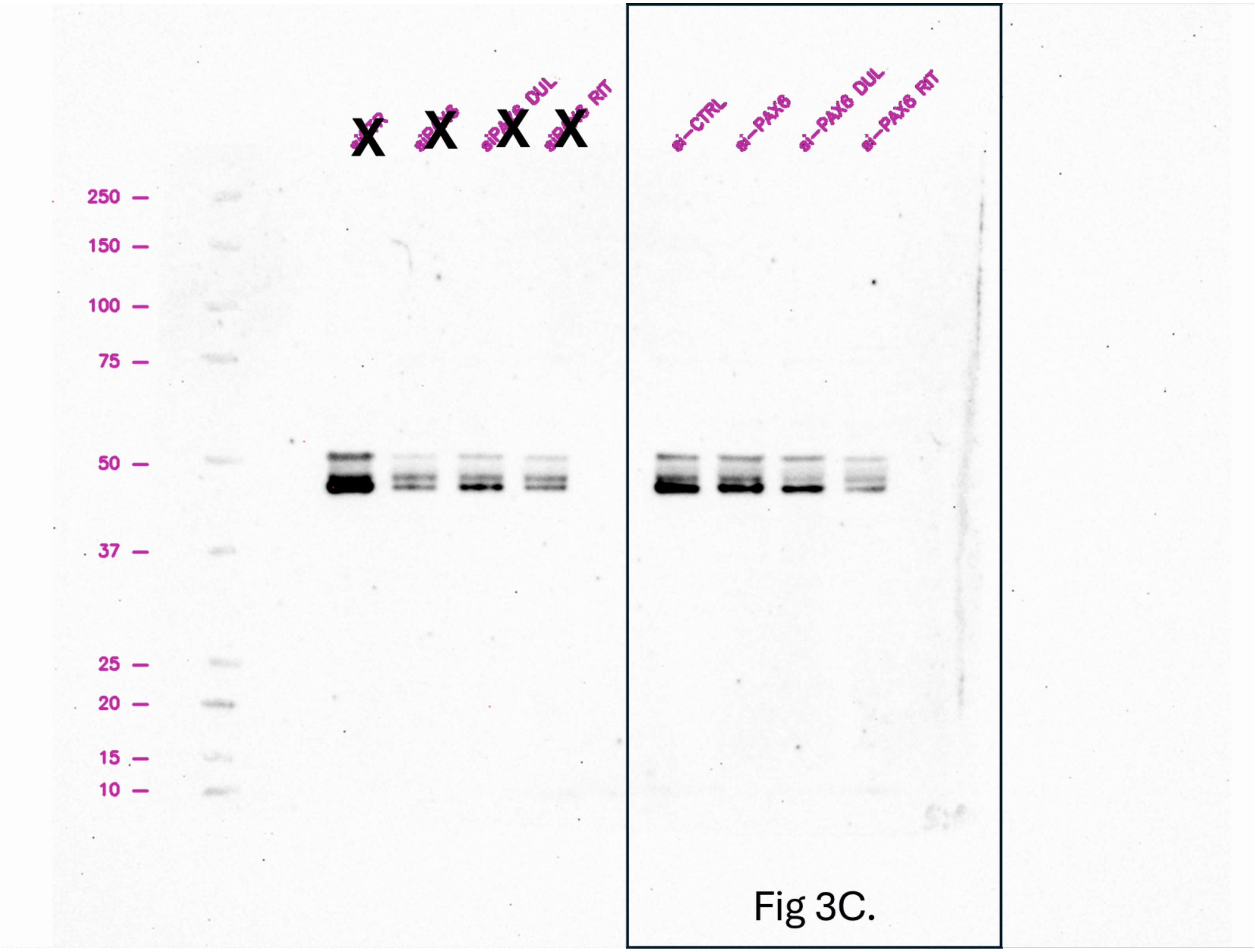

Fig 3C.

Image is captured with chemiluminiscence

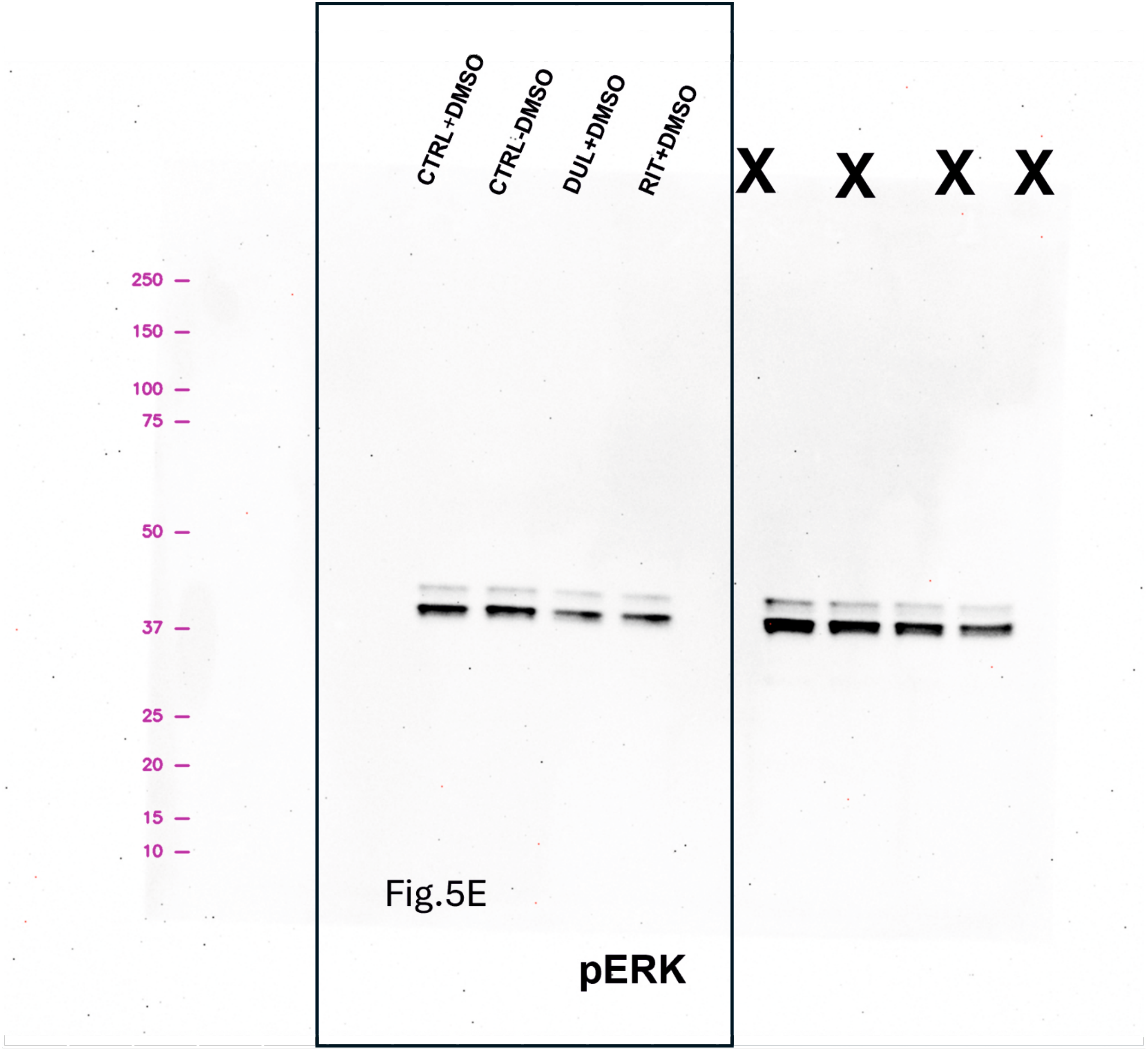

Image is captured with chemiluminiscence

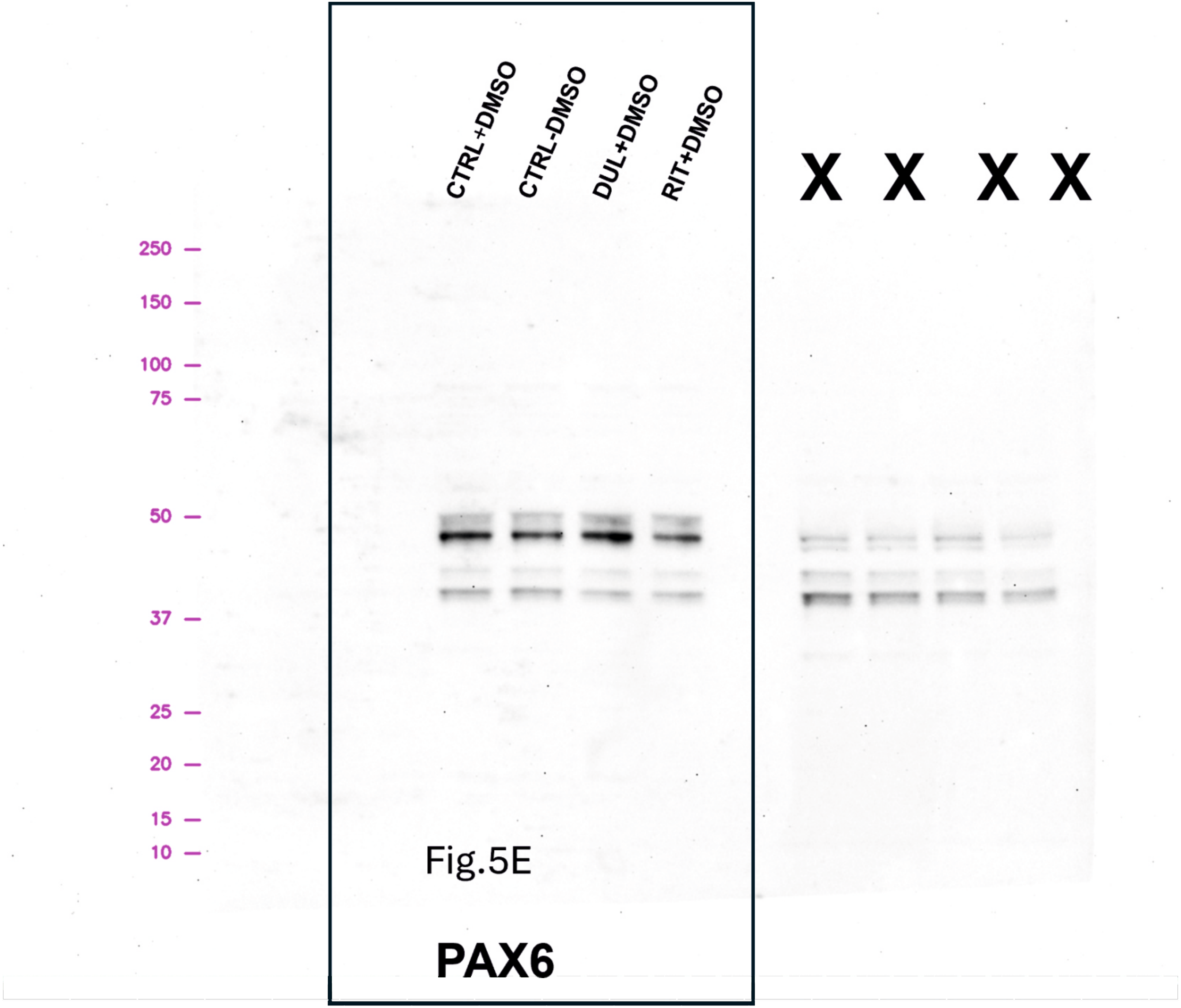

Image is captured with chemiluminiscence

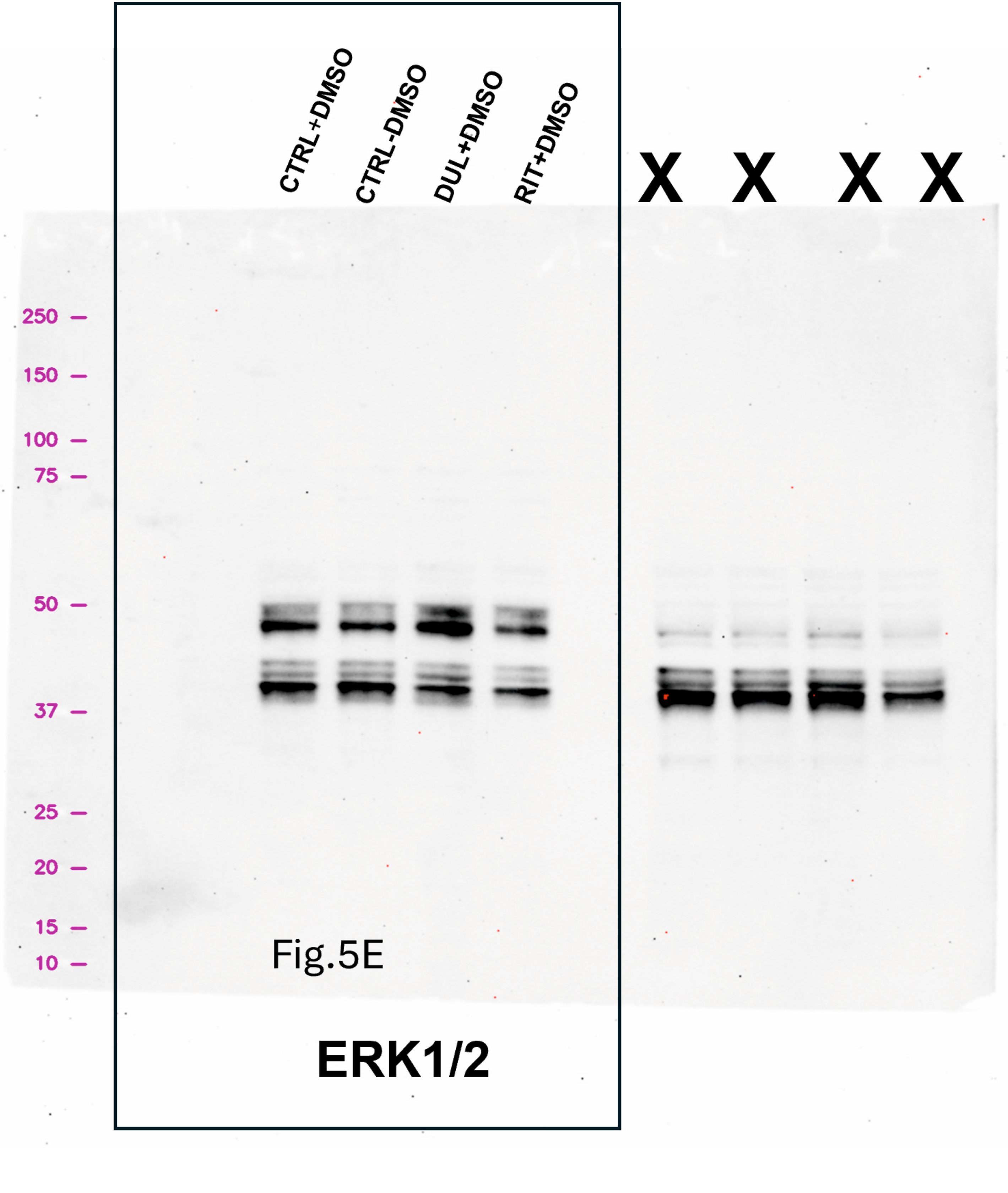

Image is captured with chemiluminiscence

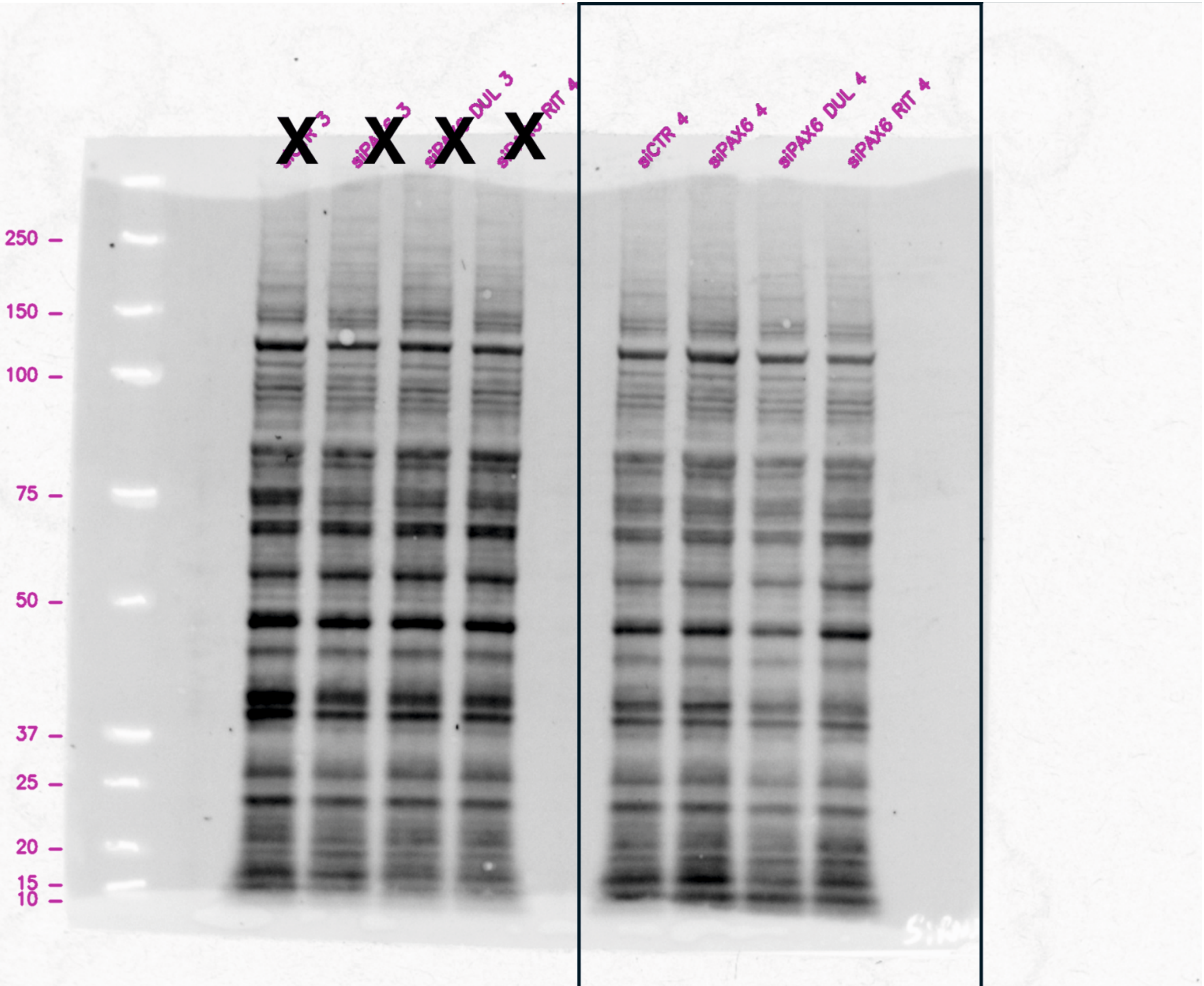

S2 Fig.

Total protein staining
